# The Mutations in *RcMYB114* Affect Anthocyanin Glycoside Accumulation in Rose

**DOI:** 10.3390/biology14030258

**Published:** 2025-03-04

**Authors:** Maofu Li, Yuan Yang, Hua Wang, Pei Sun, Shuting Zhou, Yanhui Kang, Xiangyi Sun, Min Jin, Wanmei Jin

**Affiliations:** 1 Institute of Forestry and Pomology, Beijing Academy of Agriculture and Forestry Sciences, Beijing 100093, China; limaofu2003@163.com (M.L.); yangyuanlgs@126.com (Y.Y.); happybabyhuahua@163.com (H.W.); spfate@126.com (P.S.); zhoust103@163.com (S.Z.); kk15110057556@126.com (Y.K.); 15002407391@163.com (X.S.); jinmin4710@163.com (M.J.); 2 Key Laboratory of Biology and Genetic Improvement of Horticultural Crops (North China), Ministry of Agriculture, Beijing 100093, China; 3 Beijing Engineering Research Center of Functional Floriculture, Beijing 100093, China; 4 Beijing Engineering Research Center for Deciduous Fruit Trees, Beijing 100093, China

**Keywords:** rosa, *RcMYB114* gene, artificial mutation, protein interaction

## Abstract

In plants, the R2R3-MYB transcription factors are one of the largest MYB gene families. These MYB transcription factors are very important for regulating plant growth and development. RcMYB114, RcbHLH, and RcWD40 promote anthocyanin accumulation by forming the MBW (MYB-bHLH-WD40) complex and determine the rose flower’s color. *RcMYB114* genomic sequences differ between the red petal and white varieties. Two non-synonymous substitutions were found in the open reading frame. It leads to a change in amino acids. Here, the anthocyanin content showed that there was no anthocyanin in white petals, while the anthocyanin content in red petals increased firstly at stage 2, decreased slightly at stage 4, and then increased again at stage 5. The spatiotemporal expression pattern analysis showed that *RcMYB114* was not expressed in all petals and tissues of white petals at different flower development stages. In red petal varieties, *RcMYB114* was highly expressed in petals, followed by styles, and not expressed in stems, young leaves, and stage 1 of flower development. However, *RcMYB114* has the highest expression level at the blooming stage. The *RcMYB114* sequence contains 9 SNPs in the coding region, 7 of which were synonymous substitutions that had no effect on the translation product and 2 of which were non-synonymous substitutions that resulted in amino acid alteration at positions 116 and 195, respectively. The *RcMYB114* gene in red rose was named *RcMYB114a*, and in white rose was *RcMYB114b*. *RcMYB114c* was mutated into leucine via artificial mutation; it was valine at position 116 of *RcMYB114a*, and Glycine mutated into Arginine at position 195 of *RcMYB114a* was *RcMYB114d*. *RcMYB114b* was the double mutation at positions 116 and 195 of *RcMYB114a*. The results of yeast two-hybrid experiments showed that RcMYB114a and its missense mutations RcMYB114b, RcMYB114c, and RcMYB114d could both interact with RcbHLH and RcWD40 to form the MYB-bHLH-WD40 complex. A transient transformation experiment in tobacco confirmed that *RcMYB114a* and its missense mutations *RcMYB114b*, *RcMYB114c*, and *RcMYB114d* could significantly promote the high expression of related structural genes in tobacco, together with the *RcbHLH* gene, which led to the accumulation of anthocyanins and produced the red color of the leaves. The *RcMYB114a* gene and its missense mutations *RcMYB114b*, *RcMYB114c*, and *RcMYB114d* interacted with the *RcbHLH* gene and significantly regulated the accumulation of anthocyanins. The two non-synonymous mutations of *RcMYB114* do not affect the function of the gene itself, but the content of the anthocyanins accumulated was different. This study should provide clues and references for further research on the molecular mechanism underlying the determination of rose petal color.

## Simple Summary

R2R3-MYB transcription factors constitute one of the largest MYB gene families in plants. RcMYB114, RcbHLH, and RcWD40 form the MBW (MYB-bHLH-WD40) complex, which promotes anthocyanin accumulation and determines the color of the rose flower. *RcMYB114* genomic sequences differ between the red petal and white varieties. The *RcMYB114* gene has been shown to contain two non-synonymous mutations that lead to the change of two amino acids between red and white rose varieties. In this work, the two-point mutations were generated by site-directed mutagenesis according to the sequence of the *RcMYB114* gene. These mutations resulted in significant differences in the predicted secondary and tertiary structure. Yeast two-hybrid experiments revealed that RcMYB114a and its mutant proteins RcMYB114b, RcMYB114c, and RcMYB114d could all interact with RcbHLH and RcWD40 to form the MYB-bHLH-WD40 complex. Furthermore, a transient transformation experiment in tobacco confirmed that *RcMYB114a* and its mutants *RcMYB114b*, *RcMYB114c*, and *RcMYB114d* could significantly promote the expression of related structural genes in tobacco, which resulted in the accumulation of anthocyanins and red coloring. The two non-synonymous mutations of RcMYB114 do not affect the function of the gene itself, but the content of the accumulated anthocyanins varied depending on the mutant utilized.

## 1. Introduction

One of the key phenotypic characteristics of the floral organs in ornamental plants is petal color. In an effort to speed up the selective breeding process, an increasing number of studies have been conducted, aiming to uncover the molecular and genetic causes of petal coloration. During flower development, several structural and regulatory genes involved in pigment production have been shown to impact variation in petal color patterns [1], and members of the R2R3-MYB family have been shown to play a major role in regulating this process. Rosa is a member of the genus Rosaceae, which is valued for its woody decorative blooms, cut flowers, and potted flowers. Improved characterization of the Rosa *R2R3-MYB* gene could therefore provide information about how this class of genes affects the color of flower petals. MYB transcription factors play important roles in plant development, metabolism, and responses to biotic and abiotic stresses. R2R3-MYB transcription factors have an N-terminal DNA binding domain (MYB domain) and a C-terminal activation or repression domain, and different species have been shown to have highly variable numbers of these genes. For example, 70 R2R3-MYB transcription factors were found in sugar beet [2], 108 in grape [3], 126 in Arabidopsis [4], 285 in banana [5], and 122 in Rosa [6]. Anthocyanins are primarily responsible for the red hue of petals, and their synthesis has been extensively studied. *Rosea1*, *Rosea2*, and *Venosa* genes, for example, have been shown to regulate the degree and distribution of magenta anthocyanin coloring in flowers [7]. In addition, the spatial and temporal expression pattern of *PsMYB12* in peony is closely associated with the development of petal spots [8]. The *Ruby* gene encodes a MYB transcription factor that regulates anthocyanin production, and the activity of this gene affects pigmentation in several citrus species and domesticated cultivars [9]. *NEGAN* has also been shown to be responsible for the transition to anthocyanin-pigmented petals in monkey-faced flowers [10]. Taken together, these findings indicate that activation of R2R3-MYB-related genes may be the primary driver of natural variation in the anthocyanin pigmentation of plants [7,8,9,10]. Several natural mutations exist in these genes, including single and multiple base substitutions, deletions, duplications, and insertions. Such mutations serve as a critical reservoir of biodiversity [11]. *RLL1*, *RLL2*, *RLL3*, and *RLL4* (Red Lettuce Leaves 1 through 4) are responsible for color variation in lettuce. Previous work has identified a 5-base deletion in *RLL1*, which functions as a bHLH transcription factor. This mutation abolishes its ability to stimulate anthocyanin accumulation. *RLL2* is an R2R3-MYB transcription factor, whereas *RLL3* is an R2-MYB transcription factor. *RLL3* suppresses anthocyanin production and accumulation by competing with *RLL2* for interaction with *RLL1*. In addition, a missense mutation in *RLL3* has been shown to reduce its capacity to bind *RLL1*. *RLL4* is a WD-40 transcription factor that suppresses ozone UV-B signaling. A missense mutation in *RLL4* was previously found to reduce its inhibitory effect, resulting in enhanced anthocyanin production and accumulation [12]. Missense mutations can either increase or decrease the activity of a given gene, occasionally leading to the development of distinct phenotypes. According to Li’s study, *RcMYB114* is expressed specifically in red flower organs but is not expressed in non-red varieties, such as white, yellow, and green petals [13]. The RcMYB114 protein regulates the accumulation of rosa anthocyanin by forming the MBW complex with RcbHLH and RcWD40, which ultimately influences the hue of red rosa petal pigments. A transposable element-like insertion (Rosa1) into the *RcMYB114′* promoter region causes its transcription, resulting in red petals. The *RcMYB114* genomic DNA sequence contains nine SNPs in the coding region, seven of which are synonymous substitutions and two of which are non-synonymous at positions G346T (amino acid V116L) and G586A (amino acid G195R) between red and white varieties.

Here, to determine the effect of the *RcMYB114* gene’s non-synonymous mutations on its protein structure and gene function, the non-synonymous point mutations of *RcMYB114* were generated by site-directed mutagenesis. How these non-synonymous point mutations affect anthocyanin accumulation was elucidated by combining it with molecular analysis, biochemical, and functional analyses. We cloned the genome sequence of the *RcMYB114* in the red and white rosa varieties, respectively. The coding region of the *RcMYB114* sequence in white varieties was deduced according to the genome sequence. To differentiate the two genes, we designated the *RcMYB114* gene as *RcMYB114a* (MW239569) and *RcMYB114b* (MW239570), respectively, in red and white rosa [13]. Additionally, the coding region *RcMYB114b*, *RcMYB114c*, and *RcMYB114d* mutations from the *RcMYB114a* were site-directed mutagenesis by overlap extension using the polymerase chain reaction [14]. Y2H and tobacco transient transformation experiments were then used to determine the effect of these mutations on anthocyanin accumulation. Our research provides a reference method for rapid identification of a gene mutation and assistance for accurately controlling the transcription of a target gene by the CRISPR systems.

## 2. Materials and Methods

### 2.1. Experimental Materials

We utilized the red-petal ‘Slate’s Crimson China’ (*R. chinensis*) [15], which was planted in the Rosa Germplasm Resource Garden of the Institute of Forestry and Pomology, Beijing Academy of Forestry and Pomology Sciences. The leaves, stems, styles, and petals were collected at stage 1: small bud, stage 2: large bud, stage 3: initial opening period, stage 4: blooming period, and stage 5: withering period, frozen in liquid nitrogen and stored at −80 °C. DNA was extracted according to the instructions of the Plant Genome DNA Extraction Kit (DP360, Tiangen Biotech Co., Beijing, China), and total RNA was extracted from the leaves, stems, styles, and petals at different stages of flower development using an RNAprep Pure Plant Kit (DP441, Tiangen Biotech Co., Beijing, China). Extracted RNA was then assessed for quantity and purity via gel electrophoresis.

### 2.2. Cloning of the RcMYB114 Gene and Producing the Mutations by Site-Directed Mutagenesis

Primers were designed based on the gene sequence of *RcMYB114* (Table 1), followed by cloning of the gene in ‘Slate’s Crimson China’. The cDNA of the petal was synthesized according to the instructions of the RevertAid First Strand cDNA Synthesis Kit (K1622, Thermo Scientific Inc., Waltham, MA, USA). The cDNAs were used as templates for PCR, with Li’s method employed for PCR conditions [13]. The *RcMYB114* PCR products were purified using the Spin Column DNA Gel Extraction Kit (Sangon Biotech (Shanghai) Co., Ltd., Shanghai, China) and ligated with a T vector (Tiangen Biotech Co., Beijing, China) and transformed into *Escherichia coli* to select positive clones for sequencing (Sangon Biotech (Shanghai) Co., Ltd., Shanghai, China). The plasmid DNA of *RcMYB114a*-positive clones was extracted. The *RcMYB114b*, *RcMYB114c*, and *RcMYB114d* mutations were produced by overlap extension on the PCR instrument using the plasmid DNA of *RcMYB114a* positive clones as a template because the *RcMYB114* was expressed specifically in red flower organs but was absent from non-red varieties. The *RcMYB114c* mutation was generated at position G346T (amino acid V116L), and the *RcMYB114d* mutation was generated at position G586A (amino acid G195R). The *RcMYB114b* mutation was generated simultaneously at positions G346T (amino acid V116L) and G586A (amino acid G195R). The *RcMYB114b*, *RcMYB114c*, and *RcMYB114d* PCR products were purified, ligated with a T vector, and transformed into *E. coli*. The positive clones were sequenced.

### 2.3. Gene Expression Analysis

RNA from the leaf, stem, style, and petal at various developmental stages was reverse transcribed into cDNA. For gene expression quantification, we utilized the previously synthesized cDNA as a template with *Actin (XM_024323957)* used as the internal reference gene (Table 1). Semi-quantitative PCR was carried out as previously described by Li, with a cycle number of 30 [15]. Each experiment was carried out in triplicate. The 2^−ΔΔCT^ method was used to calculate the relative expression of each gene [16]. All analyses were performed with three biological replicates. Differences in gene expression were performed with a *t*-test.

### 2.4. Yeast Vector Construction and Yeast Two-Hybrid Assay

The full-length fragments of *RcMYB114a*, *RcMYB114b*, *RcMYB114c*, and *RcMYB114d* were obtained by digesting the *RcMYB114a*, *RcMYB114b*, *RcMYB114c*, and *RcMYB114d* positive clones on the T vector using endonuclease *Nde* I and *BamH* I. The digested products *RcMYB114a*, *RcMYB114b*, *RcMYB114c*, and *RcMYB114d* were gel detected, purified, and inserted into the sites *Nde* I and *BamH* I of pGADT7 and pGBKT7 yeast vectors. The viable clones were confirmed as positive through sequencing. The plasmid DNA of positive clones was extracted. Y2H experiments were performed as previously described by Li [13]. The yeast strain AH109 was used, and the various combinations of BD and AD vectors were co-transformed using the PEG (polyethylene glycol)-mediated lithium acetate method. The co-transformants were incubated on the SD/-Leu/-Trp medium for three days at 30 °C. Colonies from the two-deficient medium were then selected and inoculated on the SD/-Trp/-Leu/-His/-Ade medium, incubated at 30 °C for seven days, and then photographed. Yeast clones growing on the same SD/-Trp/-Leu/-His/-Ade medium were stained for 30 min with 3–5 µL of 4 mg mL^−1^ X-α-gal; normal growth with a blue color was considered positive and photographed again.

### 2.5. Construction of Transient Overexpression Vector and Tobacco Transformation

The yeast plasmid DNA that contained *RcMYB114a*, *RcMYB114c*, *RcMYB114d*, *RcMYB114b*, and *RcbHLH* was used as templates for PCR amplification using full-length primers with restriction sites (Table 1). All PCR products were digested using the restriction enzymes. *Age* I and *Stu* I digested products were purified and inserted between the *Age* I and *Stu* I sites of the Hyper Trans system pEAQ-HT vector by enzymatic ligation and transformed into *E. coli*, respectively. Clones containing the correct sequence of *RcMYB114a*, *RcMYB114c*, *RcMYB114d*, *RcMYB114b*, and *RcbHLH* were then transformed into *Agrobacterium tumefaciens* GV3101, respectively, and their positive clones were selected and incubated in Luria Bertani media overnight at 28 °C. The bacterial solution was resuspended in 15 mL of exudates with acetosyringone, and the OD_600_ was adjusted to 0.2. The mixture was then injected into the leaves of *Nicotiana benthamiana*. The color change of the leaves was observed daily, and the samples were collected and measured for anthocyanin content at days four and six. The experiment was carried out with three biological replicates, with at least three plants injected for each replicate.

### 2.6. Determination of Anthocyanin Glycoside Content

The pH differential method previously described by Wang [17] was used to determine the anthocyanin content in rosa petals and the transformed tobacco leaves. The petals of Rosa and transformed tobacco leaves were ground in liquid nitrogen, followed by extraction using a 0.1% HCl-methanol solution for 4 h at room temperature in the dark. Then, the supernatant was filtered through a 0.2 μm filter membrane. The absorbance values at 510 nm and 700 nm were measured using a multifunctional microwell plate tester, and the absorbance difference was calculated to determine the content of anthocyanin glycoside. The contents of anthocyanin glycoside were calculated using anthocyanin content A = (A_510_ − A_700_) pH _1.0_ − (A_510_ − A_700_) pH _4.5_, denoted as the number of mg per 100 g fresh weight (FW).

## 3. Results

### 3.1. Anthocyanin Content of ‘Slater’s Crimson China’ Petals at Different Developmental Stages

**Figure 1:**
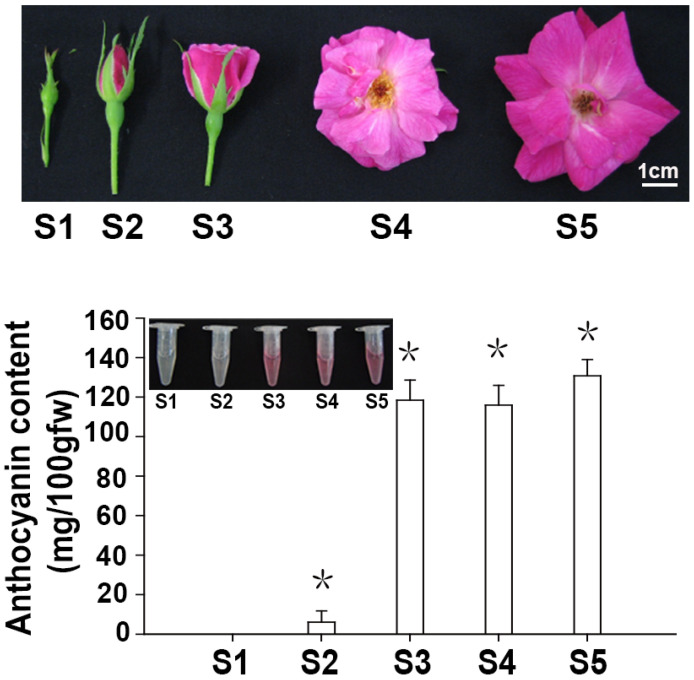
Morphology and anthocyanin content in ‘Slater’s Crimson China’ flowers at different developmental stages. Slater’s Crimson China’ flowers at five different development stages. S1: small bud stage; S2: large bud stage; S3: initial opening period; S4: blooming period; S5: withering period; Anthocyanin content in ‘Slater’s Crimson China’ flowers at the five developmental stages. Asterisks (*) represent that the values of total anthocyanin content (n = 3, ±SE) are significantly different at *p* < 0.05 as determined using independent *t*-test.

Petals of ‘Slater’s Crimson China’ at five different developmental periods are shown in Figure 1. The flowers of ‘Slater’s Crimson China’ contained 10–15 petals, which were disk-shaped, 3–5 cm in diameter, and red. Analysis of anthocyanin content for ‘Slater’s Crimson China’ petals showed that the levels initially increased, followed by a minor decrease, before increasing during the final developmental stages. The anthocyanin content was 0 in Stage 1 and increased rapidly with flower development, reaching 121.5 mg/100 g FW in Stage 3. There was a slight decrease at Stage 4, before the anthocyanin content peaked at 130 mg/100 g FW in Stage 5.

### 3.2. Expression Analysis of RcMYB114 in ‘Slater’s Crimson China’

**Figure 2:**
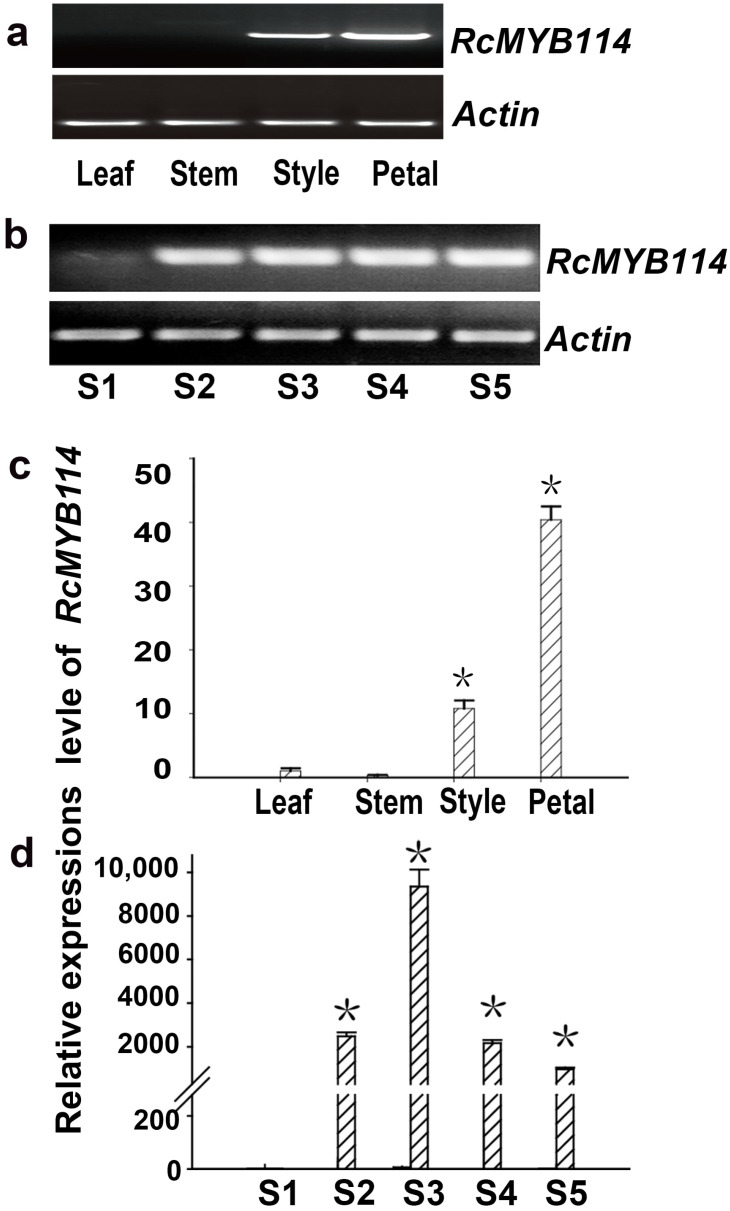
The expression pattern of *RcMYB114* in ‘Slater’s Crimson China’. (**a**,**b**): Semiquantitative RT-PCR analysis of *RcMYB114* expression in different tissues and petals at different developmental stages of ‘Slater’s Crimson China’; (**c**,**d**): *RcMYB114* expression in ‘Slater’s Crimson China’ different tissues and petals at different developmental stages determined by quantitative RT-PCR. *Actin* gene was used as the internal reference gene. Asterisks (*) represent that the values of the corresponding transcription levels (n = 3, ±SE) are significantly different at *p* < 0.05 as determined using independent *t*-test.

The expression of the *RcMYB114* gene at different flower developmental stages and in different tissues of ‘Slater’s Crimson China’ was analyzed by semi-quantitative and real-time quantitative RT-PCR. This analysis revealed that *RcMYB114* was most highly expressed in petals, followed by styles, and was not expressed at all in stems or young leaves (Figure 2a,b). In ‘Slater’s Crimson China’, *RcMYB114* was not expressed in the small bud stage of flower development; its expression level started to increase in the large bud stage, maximized in the early flowering stage, and then started to decline (Figure 2c,d).

**Figure 3:**
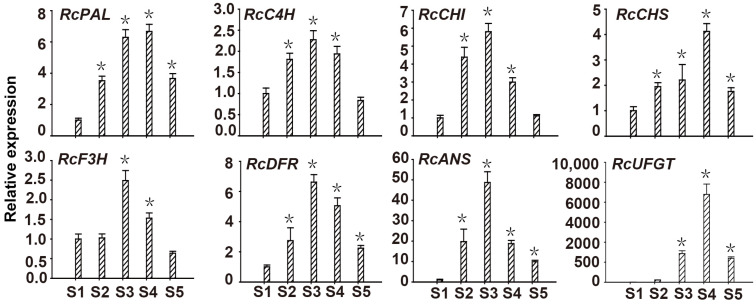
Expression of structural genes of anthocyanin biosynthesis in ‘Slater’s Crimson China’ petals at different developmental stages. *PAL*: phenylalanine ammonia-lyase, *C4H*: cinnamate 4-hydroxylase, *CHI*: chalcone isomerase, *CHS*: chalcone synthase, *F3H*: flavanone 3-hydroxylase, *DFR*: dihydroflavonol-4-reductase/flavanone-4-reductase, *ANS*: anthocyanidin synthase, *UFGT*: flavonol-O-glucosyltransferases. Asterisks (*) represent that the values of the corresponding transcription levels (n = 3, ±SE) are significantly different at *p* < 0.05 as determined using independent *t*-test.

To investigate the effect of *RcMYB114* on structural genes phenylalanine ammonia-lyase (*PAL*), cinnamate 4-hydroxylase (*C4H*), chalcone synthase (*CHS*), chalcone isomerase (*CHI*), flavanone 3-hydroxylase (*F3H*), dihydroflavonol-4-reductase flavanone-4-reductase (*DFR*), anthocyanidin synthase (ANS), and flavonol-O-glucosyltransferases (*UFGT*) in the anthocyanin pathway, we conducted real-time quantitative RT-PCR of structural genes during different petal developmental periods in ‘Slater’s Crimson China’. Results showed that *RcMYB114* expression in ‘Slater’s Crimson China’ was correlated with the expression of *DFR*, *ANS*, and *UFGT*, suggesting that *RcMYB114* may be involved in the regulation of anthocyanin accumulation (Figure 3).

### 3.3. Cloning and Sequence Analysis of RcMYB114 in ‘Slater’s Crimson China’

**Figure 4:**
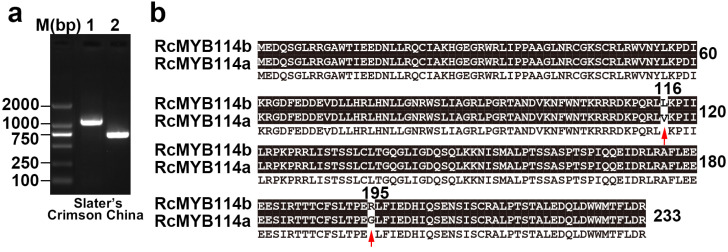
Cloning of *RcMYB114* in ‘Slater’s Crimson China’ and alignment of the deduced proteins. (**a**). Cloning of *RcMYB114* in ‘Slater’s Crimson China’ using genomic DNA (1) and cDNA (2) as templates; (**b**). Alignment of RcMYB114 proteins from ‘Slater’s Crimson China’. The red arrow indicates the difference in amino acids caused by two non-synonymous. The protein sequence RcMYB114a was cloned from red petal cDNA, and RcMYB114b was calculated from the genomic sequence *RcMYB114* in white petal variety.

Primers for amplification of the full-length *RcMYB114* gene in ‘Slater’s Crimson China’ were designed based on the genomic sequence of *RcMYB114* (accession no: MW239568) and used to amplify the genomic DNA and cDNA. Amplification of the genomic DNA and cDNA of ‘Slater’s Crimson China’ resulted in a band of ~1200 bp and 700 bp (Figure 4a). Consistent with the expression data, *RcMYB114* was amplified in the petal cDNA sample of ‘Slater’s Crimson China’. Because *RcMYB114* could not be expressed in white petal cDNA [13], we deduced its coding sequence (CDS) and the encoded protein by removing introns from its genomic sequence based on the *RcMYB114* gene model in ‘Slater’s Crimson China’. A comparison of the predicted proteins revealed that there are two amino acid differences at positions 116 and 195. We designated the *RcMYB114* allele in the red variety as *RcMYB114a* and that in the white variety as *RcMYB114b* (Figure 4b).

Biochemical properties between the RcMYB114 and its mutants were slightly different (Supplementary Table S1). The RcMYB114a, RcMYB114b, RcMYB114c, and RcMYB114d proteins had molecular weights of 26.76, 26.87, 26.77, and 26.86 kD, respectively. RcMYB114a and RcMYB114c share the same isoelectric point, total number of negatively charged residues, and grand average of hydropathicity. Similarly, these parameters for RcMYB114b and RcMYB114d are the same. RcMYB114a and RcMYB114d share the same instability index and aliphatic index. Similarly, these two parameters for RcMYB114b and RcMYB114c are the same.

**Figure 5:**
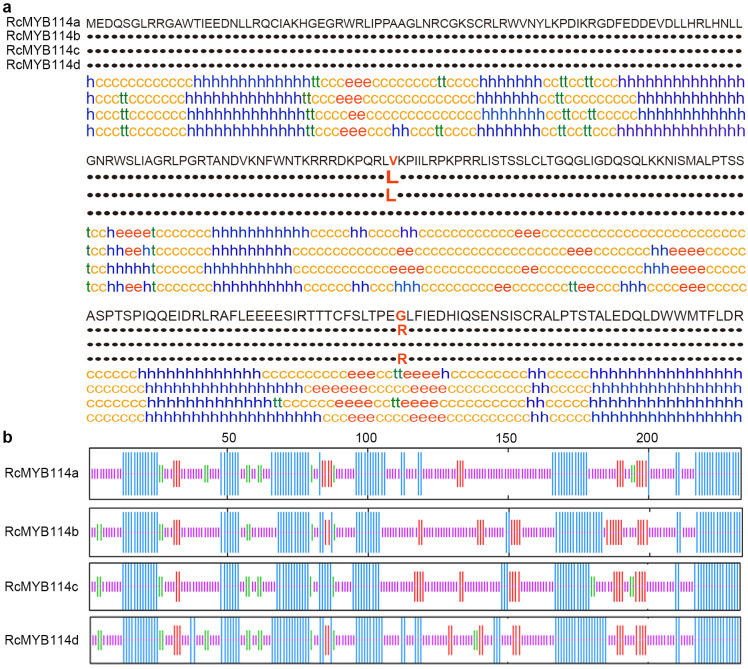
Comparison of the RcMYB114a and its mutant’s structures. (**a**,**b**). Predicted secondary structures of RcMYB114a, RcMYB114b, RcMYB114c, and RcMYB114d, in (**a**), the c: Random coil; e: Extended strand; h: α-helix; t: Beta turn; in (**b**), the pink module: Random coil; red module: Extended strand; blue module: α-helix; green module: Beta turn.

Also, the predicted secondary structures of the proteins are different between the RcMYB114a and its mutants (Supplementary Table S2). The RcMYB114a, RcMYB114b, RcMYB114c, and RcMYB114d proteins’ random coil accounts for 51.93%, 51.07%, 50.21%, and 45.06%; the α-helix 35.62%, 35.19%, 35.19%, and 40.77%; extended strand 7.30%, 10.30%, 8.58%, and 8.15%; β turn 5.15%, 3.43%, 6.01%, and 6.01%, respectively. Overall, the secondary structures of RcMYB114a, RcMYB114b, RcMYB114c, and RcMYB114d differed due to the two mutations identified earlier (Figure 5a,b).

### 3.4. RcMYB114a and Its Mutations Can Interact with RcbHLH and RcWD40

**Figure 6:**
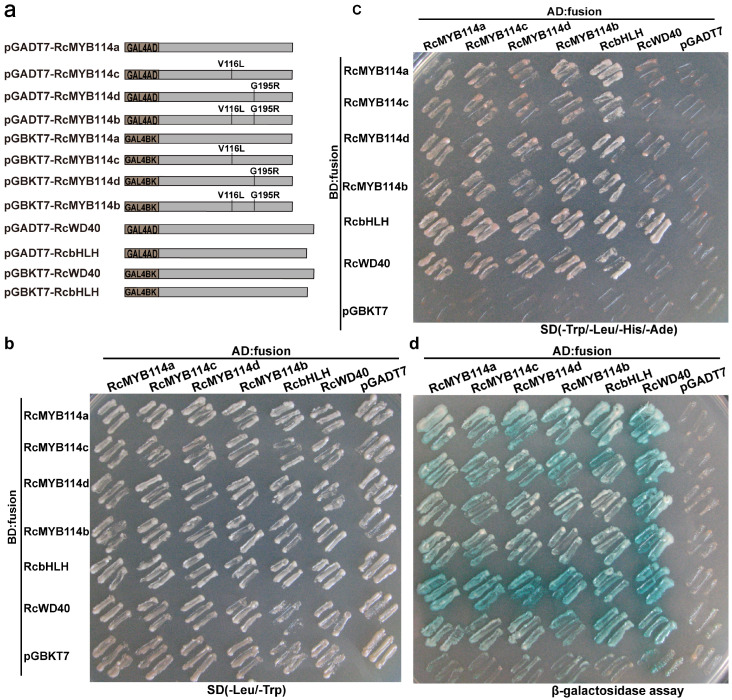
Interaction of different RcMYB114 variants with RcWD40 and RcbHLH assessed by yeast two-hybrid (Y2H). (**a**). Schematic diagram of different vectors used for the Y2H experiment; (**b**). The co-transformants were incubated on SD/-Leu/-Trp plate; (**c**). The co-transformants were incubated on SD/-Trp/-Leu/-His/-Ade plate; (**d**). β-galactosidase tests were performed on the same SD/-Trp/-Leu/-His/-Ade plate, and positive clones were dyed by using 3–5 µL 4 mg/mL X-α-gal, and false-positive activation was excluded using the P53 plus SV40 vector.

R2R3 MYB transcription factors are known to regulate the synthesis and accumulation of anthocyanin by interacting with bHLH and WD40 to form the MYB-bHLH-WD40 complex [8,17,18,19,20,21,22,23,24]. To investigate whether the two mutations in *RcMYB114* affect its interaction with bHLH and WD40, we performed yeast two-hybrid (Y2H) experiment. The RcMYB114b (V116L and G195R), RcMYB114c (V116L), and RcMYB114d (G195R) mutation sequences were generated by site-directed mutagenesis from the ‘Slater’s Crimson China’ *RcMYB114* CDS (Figure 6a). Then, the mutation products of RcMYB114b, RcMYB114c, and RcMYB114d from RcMYB114a were cloned into the pGADT7 and pGBKT7 vectors, respectively. Results showed that all four forms of RcMYB114 could interact with both RcbHLH and RcWD40 to form the MYB-bHLH-WD40 complex (Figure 6b–d), suggesting that the two amino acid substitutions in RcMYB114 do not significantly affect protein function.

### 3.5. Effect of RcMYB114 and Its Mutation on Anthocyanin Accumulation

**Figure 7:**
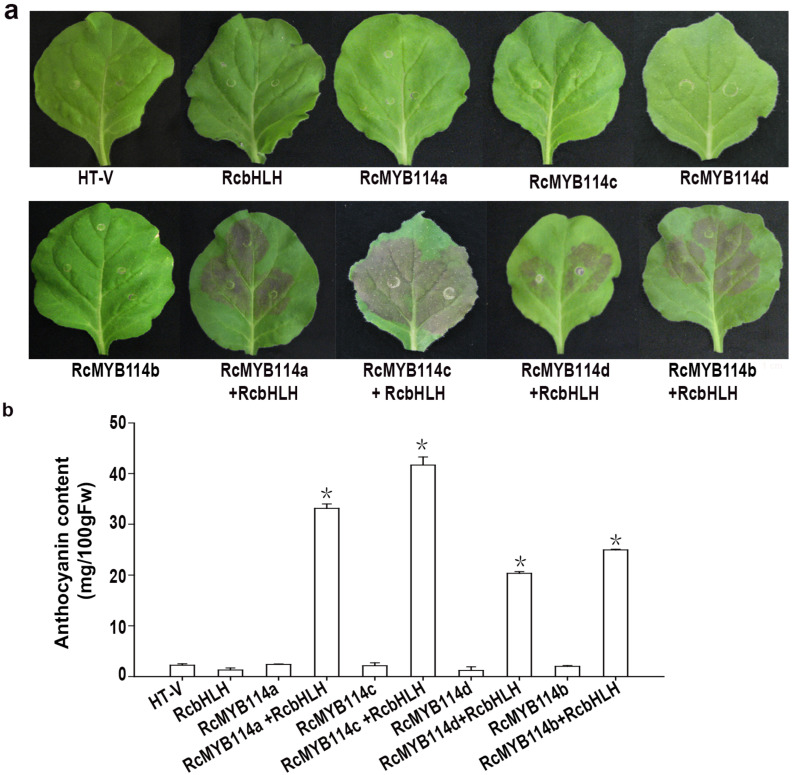
Morphologyand anthocyanin content of tobacco leaves transformed with different *RcMYB114* alleles. (**a**): Phenotype of tobacco leaves after injection of plasmids containing different *RcMYB114* alleles; (**b**): Assays of the anthocyanin contents of tobacco leaves that overexpressed different *RcMYB114* alleles. Assays were carried out with three biological replicates, with at least three plants injected for each replicate. Asterisks (*) represent that the values of total anthocyanin content (n = 3, ±SE) are significantly different at *p* < 0.05 as determined using independent *t*-test.

To further test the effect of *RcMYB114* and its mutants on anthocyanin production in vivo, *RcMYB114a*, *RcMYB114b*, *RcMYB114c*, and *RcMYB114d* were transiently overexpressed into tobacco leaves. The transformed leaves were found to accumulate a significantly higher level of anthocyanin than the empty vector control, indicating that all four *RcMYB114* alleles are able to increase anthocyanin levels in vivo (Figure 7a). These results are consistent with those of the Y2H experiment. In addition, there were differences in the level of anthocyanin content in the injected leaves and the strength of leaf coloration. The anthocyanin content was 33.11, 24.29, 41.68, and 20.35 mg/100g FW 10~20 fold of the empty vector control in RcMYB114a, RcMYB114b, RcMYB114c, and RcMYB114d transgenic leaves, respectively. These findings indicate that the point mutation in the *RcMYB114* gene has an impact on anthocyanin synthesis and accumulation. The mutation of *RcMYB114c* had the greatest effect, while the mutation in *RcMYB114d* had the least. More importantly, we found that the transformation of *RcMYB114a*, *RcMYB114b*, *RcMYB114c*, and *RcMYB114d* alone did not lead to obvious anthocyanin accumulation in tobacco leaves, suggesting that these genes function corporately with *RcbHLH* to produce anthocyanin (Figure 7a,b).

## 4. Discussion

### 4.1. The RcMYB114 Gene Is a Key Regulator of Anthocyanin Accumulation in Rose

Anthocyanins are important secondary metabolites with multiple biological functions [25]. Members of the R2R3-MYB transcription factor family have been shown to regulate the anthocyanin biosynthetic pathway and thus impact anthocyanin biosynthesis and accumulation [8,17,18,19,26,27,28,29,30]. Although different *R2R3-MYB* genes control the color of fruits and flowers, their regulatory mechanisms are very similar. For example, the *an2* allele is defective in *an2* function due to a base insertion that resulted in a frameshift, thus producing the white petal color [31]. In *Antirrhinum majus*, three different alleles of an *R2R3-MYB*, *Rosea1*, *Rosea2*, and *Venosa*, have been shown to work together to regulate the intensity and pattern of magenta anthocyanin pigmentation in petals [7]. In this system, wild-type petals are almost entirely colored, and the corolla contains high concentrations of magenta anthocyanins. However, two different mutant alleles of the *Rosea* locus with indels (*ros^col^* and *ros^dor^*) result in reduced levels of anthocyanin on the inner epidermis of the petals, the base of the tube, and the outer epidermis on the dorsal surface of leaves [7]. In chrysanthemum petals, the transient overexpression of *CmMYB9a*, which belongs to subgroup 7 of the R2R3-MYB family, increases the transcript levels of anthocyanin and flavonoid-related genes, leading to the accumulation of anthocyanins and flavonoids [32]. Different *R2R3-MYB* genes display different spatiotemporal expression patterns, leading to the coloration of different parts of petals. For example, in *Clarkia gracilis* petals, *CgsMYB12* determines the synthesis and accumulation of anthocyanins mainly at the base, *CgsMYB1* controls the coloration of the spots, whereas *CgsMYB11* and *CgsMYB6* regulate the accumulation of background pink pigmentation [33]. *LhMYB6* and *LhMYB12* have also been shown to positively regulate anthocyanin biosynthesis in lily (*Lilium* spp.) petals, and co-expression of these two genes can activate the expression of anthocyanin biosynthesis-related genes in lily bulbs [34]. *LhMYB12* mainly regulates the synthesis and accumulation of anthocyanins in lily petals, filaments, and styles, whereas *LhMYB6* is associated with spot- and light-induced anthocyanin synthesis in the perianth [34,35,36,37,38]. The appearance of petal spots in lilies is mainly caused by an allele of *LhMYB12*, *LhMYB12-Lat*, which promotes petal anthocyanin accumulation [39]. The R2R3-MYB protein scan regulates anthocyanin synthesis either alone or by interacting with bHLH and WD40 to form the MBW complex [8,17,18,19,20,21,22,23,24]. In addition, the maize *ZmPAC1* gene (WD40) can interact with *ZmR1* (bHLH) and *ZmC1* (MYB) to jointly control anthocyanin biosynthesis [20]. Ben-Simhon et al. found that the pomegranate *PgWD40* can be co-expressed with *PgAn1* (bHLH) and *PgAn2* (MYB) to regulate the expression of anthocyanin structural genes *DFR* and *LDOX* [40]. MYB and bHLH transcription factors can still interact with each other without the presence of WD40 to regulate the expression of anthocyanin synthesis genes. For example, the interaction in radish (*Raphanus sativus* L.) between a bHLH transcription factor RsTT8 and RsMYB1 significantly increased the expression levels of endogenous anthocyanin biosynthetic genes and led to anthocyanin accumulation [41]. Co-expression of *MrbHLH1* and *MrMYB1* in poplar and *LcbHLH3* and *LcMYB1* in litchi (*Litchi chinensis* Sonn.) both activate downstream anthocyanin biosynthesis genes and promote anthocyanin synthesis in fruits [42,43]. Previous studies find that the *RcMYB114* genomic DNA sequences were normal both in red and white flower organs, but there are significant differences at the cDNA level between the red and white flowers. *RcMYB114* is expressed specifically in red flower organs but is not expressed in non-red varieties, such as white, yellow, and green petals. The *RcMYB114* genomic DNA sequences contain nine SNPs in the coding region, seven of which are synonymous substitutions and two of which are non-synonymous between red and white varieties. In this study, transient co-expression of *RcMYB114* and *RcbHLH* rather than overexpression of *RcMYB114* alone in tobacco leaves was able to activate downstream anthocyanin biosynthesis genes and promote anthocyanin accumulation. However, there are differences in anthocyanin content between the *RcMYB114a* and its mutants, which may be caused by differences in the protein structure of the gene due to the point mutations. Taken together, these findings indicate that the *RcMYB114* gene controls the coloration of rose petals by working in concert with *RcbHLH*.

### 4.2. Differential Activity of Genes with Missense Mutations

The point mutations in structural or regulatory genes can either attenuate or promote end-product accumulation. For example, the missense mutation in an R2R3-MYB in green lettuce, *RLL3*, caused a tryptophan-to-serine substitution at position 52 and resulted in the inability of the RLL3 protein to bind RLL1 (bHLH), thereby inhibiting anthocyanin biosynthesis and accumulation and producing green lettuce [12]. The missense mutations also exist in key genes involved in the phenylpropanoid pathway, such as the missense mutation C4H4 position 108 threonine to alanine, which was significantly associated with the differential accumulation of flavonoids, anthocyanins, and proteins. The histidine to glutamine missense mutation at position 438 of *HQT* was associated with changes in flavonoids and chlorogenic acid accumulation, and the valine to alanine missense mutation at position 65 of *ANS1* was associated with the differential accumulation of anthocyanins and sugars [44]. In addition, the barley mutant ant 18–162 produces anthocyanin-free barley due to a missense mutation in the *DFR* structural gene, resulting in a mutant lacking DFR activity [45]. In Arabidopsis, the *mvs1* (methylviologen-sensitive) mutant can increase unsaturated FA abundance and over-accumulated ROS caused by a missense mutation (G1349 substituted as A) of a cytochrome P450 *CYP77A4* gene [46]. In this study, we found that either a single mutation or two simultaneous mutations in the RcMYB114 protein impacted its ability to activate the expression of downstream anthocyanin biosynthesis genes in tobacco, resulting in differential anthocyanin accumulation. Simultaneously, our study provides a reference for accurately controlling the transcription of one gene by using the CRISPR method.

## 5. Conclusions

The *RcMYB114* gene has been shown to contain two non-synonymous mutations that lead to the change of two amino acids between red and white rose varieties. These two non-synonymous mutations resulted in significant differences in the predicted secondary and tertiary structures. The two-point mutations were generated by site-directed mutagenesis according to the sequence of the *RcMYB114* gene. Yeast two-hybrid experiments and transient transformation experiments in tobacco revealed that RcMYB114a and its mutant proteins RcMYB114b, RcMYB114c, and RcMYB114d could interact with RcbHLH and RcWD40 to form the MYB-bHLH-WD40 complex and could significantly promote the expression of related structural genes, which resulted in the accumulation of anthocyanins and red coloring. The two non-synonymous mutations of *RcMYB114* do not affect the function of the gene itself, but the content of the accumulated anthocyanins varied depending on the mutant utilized.

## Figures and Tables

**Table 1:** Primer sequences.

Gene Name	Purpose	Primer Sequence (5′→3′)
*RcMYB114a*	Gene clone	F: CAACGTCATTAACTGTGGGATC
		R: GCCGTGAGCAGTGGCTTTC
*RcMYB114a*, *RcMYB114b*, *RcMYB114c*, and *RcMYB114d*	Transient expression	F:GAAATTtctagaATGGAGGACCAGTCGGGTTTGAG
		R: GAAATTgagctcTCATTATCGATCTAAGAATGTCATCC
*RcbHLH*		F:GAAATTtcgcgaATGGCTACACCGCCACCGAGTAGTAGC
		R: GAAATTaccggtTTAAGAGTCAGATTGGGGTATCAC
*RcMYB114c*	site-directed mutagenesis	F: CCACAAAGATTGTTGAAGCCCATCATAC
		R: AGTATGATGGGCTTCAACAATCTTTGTG
*RcMYB114d*		F: TTGACCCCTGAAAGATTATTTATTGAAG
		R: ATCTTCAATAAATAATCTTTCAGGGGTC
*RcMYB114a:AD*, *RcMYB114b:AD*, *RcMYB114c:AD*, and *RcMYB114d:AD*	Yeast two-hybrid assays	F: GAAATTcatatgATGGAGGACCAGTCGGGTTTGAG
		R: GAAATTgaattcTCATTATCGATCTAAGAATGTCATCC
*RcMYB114a:BK*, *RcMYB114b:BK*, *RcMYB114c:BK*, and *RcMYB114d:BK*		F: GAAATTcatatgATGGAGGACCAGTCGGGTTTGAG
		R: GAAATTgaattcTCATTATCGATCTAAGAATGTCATCC
*RcWD40:AD* and *RcWD40:BK*		F: GAAATTcatatgATGGAGAACTCGACCCAAGAATC
		R: GAAATTgaattcTCAAACCTTCAACAGCTGCATCTTA
*RcbHLH:AD* and *RcbHLH:BK*		F: GAAATTggatccATGGCTACACCGCCACCGAGTAGTAGC
		R: GAAATTgaattcTCATTAAGAGTCAGATTGGGGTATCAC
*RcMYB114a*	RT-qPCR	F:ACCAAGCGGCGTCGGGACAAAC
		R: CCCGTCAAACAGAGTGAACTGGTCG
*RcC4H*		F:ATGTTCGACAGGAGATTTGAAAGCG
		R:ATTATACTCGAAGCTCTGCGCCAAC
*RcCHI*		F:TTTCCTCCCGCCGTCAAGCC
		R:CCAAGTAGACTCCAATCGCCGTGAA
*RcCHS*		F:CTACTTTCGTATCACCAACAGCG
		R:TTCAGTCAAATACATATAACGCTTC
*RcF3H*		F:GCTCCAGGACCAAGTCGGTGGACT
		R:TGATCGGCGTTCTTGAACCTCCC
*RcANS*		F:GGAACTTGCCCTCGGCGTGG
		R:ATGACGATGGAGTTGGGCACGC
*RcPAL*		F:AAGATTTTGCGAGAAGGATTTGC
		R:GCATTCTTCTCATTCTCACCATTTGT
*RcUFGT*		F:GCCCCAAACACCCTCTTCTCA
		R:CCTGAGGCTTACCCACAAAAACA
*RcDFR*		F:CACCGTGCGAGACCCTGCTAA
		R:TCAAAATCCATAGGAGTGGCGACA
*RcActin*		F: GGCTGTTCTTTCCCTCTATGC
		R:GCGTTTCAGATGCCCAGAA

Note: F: forward primer; R: reverse primer. Lowercase letters indicate restriction sites.

## Data Availability

The original contributions presented in this study are included in the article/Supplementary Materials. Further inquiries can be directed to the corresponding author.

## Supplementary Materials

The following supporting information can be downloaded at: https://www.mdpi.com/article/10.3390/biology14030258/s1, Table S1. Biochemical properties of RcMYB114 and its mutants. Table S2. Protein secondary structure predictions of RcMYB114 and its mutants.
