# Supplementary material for: The Mutations in RcMYB114 Affect Anthocyanin Glycoside Accumulation in Rose
Source: Biology (Basel). 2025 Mar 4;14(3):258. doi: 10.3390/biology14030258 (PMC11939692; doi:10.3390/biology14030258)
Supplement: Supplementary file 1 [file biology-14-00258-s001.zip › biology-3471683-supplementary.pdf]

Supporting information

Table S1. Biochemical properties of RcMYB114 and its mutants

Protein name	AALength	MW	pI	Asp（-）+ Glu（-）	Arg（+）+ Lys（+）	Instability index	Aliphatic aa index	Hydropathicity
RcMYB114a	233	26.76	9.15	30	36	72.04	86.70	-0.61
RcMYB114b	233	26.87	9.29	30	37	71.80	87.12	-0.63
RcMYB114c	233	26.77	9.15	30	36	71.80	87.12	-0.61
RcMYB114d	233	26.86	9.29	30	37	72.04	86.70	-0.63

Table S2. Protein secondary structure predictions of RcMYB114 and its mutants

Protein name	AA Length	random coil(%)	α-helix(%)	extended strand(%)	β turn(%)
RcMYB114a	233	51.93	35.62	7.30	5.15
RcMYB114b	233	51.07	35.19	10.30	3.43
RcMYB114c	233	50.21	35.19	8.58	6.01
RcMYB114d	233	45.06	40.77	8.15	6.01
